# Trauma-Informed Support in a Dementia Helpline: Retrospective Mixed Methods Study

**DOI:** 10.2196/71746

**Published:** 2026-07-29

**Authors:** Eileen Harkess-Murphy, Rhoda Macrae, Margaret Brown, Jennifer Hall

**Affiliations:** 1 Alzheimer Scotland Centre for Policy and Practice School of Health and Life Sciences University of the West of Scotland Paisley United Kingdom; 2 Diabetes UK Glasgow, Scotland United Kingdom

**Keywords:** trauma-informed, psychological trauma, telephone helpline, dementia, Alzheimer

## Abstract

**Background:**

People with dementia and their caregivers experience significant psychological distress, which may increase their vulnerability to trauma across the dementia diagnostic and caregiving trajectory. Specialist dementia helplines offer immediate emotional support, information, and signposting; however, little empirical evidence exists about how call handlers account for potential trauma in their responses.

**Objective:**

This study aimed to examine the extent to which call handlers’ responses on the United Kingdom’s only 24-hour dementia helpline reflected trauma-informed (TI) principles of safety, trust, choice, collaboration, and empowerment and to describe caller characteristics and reasons for contact during the COVID-19 pandemic.

**Methods:**

No participants were actively recruited. Instead, the study analyzed 198 anonymized, routinely collected helpline call logs (out of 200 randomly selected) drawn from 7357 calls received by Alzheimer Scotland’s 24-hour helpline between April 2020 and April 2021. A retrospective deductive framework analysis mapped narrative summaries to 5 TI principles (safety, trustworthiness and transparency, choice, collaboration, and empowerment). Double‑coding and calibration were undertaken in line with established guidance on intercoder reliability for qualitative research. Descriptive statistics summarized caller characteristics and theme frequencies; no inferential testing was conducted due to the exploratory nature of the analysis, sample properties, and the subjective nature of theme ratings.

**Results:**

Most calls were made during daytime hours (159/198, 79.5%) and were made by carers, family members, or friends (n=179, 89.5%). Emotional support was the most frequently recorded reason for contact (91 instances), followed by carer stress (66 instances) and information on caring (51 instances). Across call handlers’ responses, collaboration (126/179, 70.4%) and empowerment (108/179, 60.3%) were the most frequently observed TI principles, followed by safety (105/179, 58.7%), choice (66/179, 37.4%), and trust (56/179, 31.3%). Safety-focused responses were more prevalent in nighttime calls than daytime calls (76.9% vs 56%). Illustrative call log excerpts demonstrated empathetic listening, validation, shared problem-solving, and signposting practices aligned with TI principles.

**Conclusions:**

In this exploratory retrospective evaluation, responses from a national dementia helpline commonly reflected TI principles, despite call handlers receiving primarily awareness-level TI content within their wider role preparation. Findings should be interpreted as descriptive and hypothesis-generating because they are based on call log summaries rather than recorded interactions and were obtained within the unique context of the COVID-19 pandemic. The results suggest that TI principles may be feasible and relevant in dementia helpline services and highlight the potential value of more structured approaches to TI workforce development. Future prospective research incorporating richer data sources such as recorded calls and call-reported outcomes is warranted to support service development and evaluation.

## Introduction

Dementia is a complex, life-altering condition characterized by progressive cognitive decline [1] that currently affects an estimated 55 million people worldwide [2]. In the United Kingdom, nearly 1 million individuals are on a dementia diagnosis pathway, while many more experience symptoms that disrupt their lives but have not yet sought medical or social support [3]. Dementia places significant emotional, psychological, and practical demands on people living with dementia and the family members who support them [4]. The process of recognizing symptoms, navigating assessment pathways, and adapting to the implications of a long-term neurodegenerative disease can be profoundly distressing. This highlights the relevance of a trauma-informed (TI) perspective, which assumes that distress may be present even if trauma has not been formally assessed [5]. Trauma is understood in this context to be “an event, series of events, or set of circumstances, that is experienced by an individual as physically or emotionally harmful or life-threatening and has lasting adverse effects on the individual functioning and mental, physical, social, emotional or spiritual well-being” [5]. Although traumatic experiences are common, not everyone who experiences them develops trauma. Similarly, while living with dementia and caring for someone with dementia is recognized as stressful, not every individual affected by dementia will experience trauma. People living with dementia and their carers will, of course, experience both good and bad days [6], alongside recognized positive outcomes such as strengthened family bonds, mutuality, a sense of purpose, and personal growth [7]. Nonetheless, individuals frequently describe stressful and frightening experiences that may overwhelm their coping capacity [8,9], highlighting the potential for distress in the dementia journey [10] and subsequent risk of trauma. By assuming a precautionary stance that high-risk populations such as people with dementia and their carer’s may be affected by trauma, we promote sensitivity and awareness, reducing the risk of further harm in practice settings [4].

Addressing the impact of trauma is an international public health priority [2], and it is important to recognize that interactions can either promote safety and recovery or contribute to retraumatization. TI approaches recognize the widespread prevalence and effect of trauma and respond to it in a manner that supports recovery, respect, trust, safety, and empowerment [5,11]. The intersection between trauma and dementia has the potential to occur before diagnosis, from symptom onset, during assessment, or after diagnosis as individuals and their carers live with dementia [12,13]. A life-limiting and terminal diagnosis such as dementia requires significant psychological and social adjustment for the entire family, but for carers, this experience is especially challenging as they also experience grief, distress, and loss while absorbing the everyday caring responsibilities, usually without expert knowledge and support [12,13].

A paradigm shift toward TI ideology embedded within practice settings in health, social care, and education has received recognition within best practice frameworks [14,15]. These are designed to mitigate the potential for activation or reactivation of vicarious and/or historical trauma [16]; however, despite TI principles featuring within national and international policy strategies, there is a dearth of evidence-based literature that explores TI approaches within practice settings [17]. This is relevant to people receiving care and families and staff involved in delivering care and support for dementia [18], who themselves are at risk of trauma as the hidden cumulative effect of psychological trauma reduces the threshold for stress tolerance [19-21]. Responding to the complex and nuanced health and social care needs of people with dementia and their carers is a growing global concern and public health challenge; current service provision has been criticized as uncoordinated, fragmented, and unfit for purpose, with inadequate signposting to available support services [22-25]. This drives a need for postdiagnostic support, signposting, and coordination that is currently inadequate and unmet on an international scale [24]. The implications of this exacerbate the distress faced by people living with dementia and their families as they attempt to understand and live with this life-changing condition and navigate support and services.

Bridging a gap in prediagnostic and postdiagnostic provision, specialist dementia helplines provide a critical lifeline at some of the most difficult, confusing, and devastating times in the lives of people impacted by dementia [26-28]. The helpline model emerged in the 1950s in the context of suicide prevention [29] and has since become a valuable resource across a wide range of public health domains, including specialist services for specific conditions such as dementia [30,31]. The immediate availability of information, practical advice, and emotional support provided by dementia helplines has been reported to alleviate psychological distress, improve well-being, and help prevent care breakdown. These benefits may reduce urgent care admissions and provide measurable mental health benefits for caregivers [28,30,32,33].

During daytime hours, various dementia specialist helplines are available for information and support across the United Kingdom; however, outside normal opening hours (eg, 9 AM-5 PM), calls are rerouted to the Alzheimer Scotland helpline. As the United Kingdom’s only 24-hour dementia helpline, it is open to residents across the United Kingdom, offering a first-level response to callers needing general information and signposting and is a gateway to other sources of prediagnostic and postdiagnostic support [34]. The helpline is a nonclinical resource staffed by volunteer call handlers who complete a 4-day program on call handling and 2-day training per year thereafter. This training includes TI content around raising the general awareness of trauma, with no specific TI practice training provided. Call handlers are unpaid yet often have extensive professional and/or lived experience of dementia and receive these calls within their own home using the telephone, without recording equipment. Although it is common practice for helplines to record calls, the Alzheimer Scotland helpline does not have this capability and instead relies on a call log form completed by the call handler.

COVID-19 (2020-2021) exacerbated global health inequalities; however, for people in existing vulnerable situations, such as people with dementia and their carers, there was an inordinate impact across multiple domains [35-37]. As existing care infrastructure underwent rapid reorientation and informal community support ceased, the Alzheimer Scotland helpline experienced a 30% increase in the number and length of calls [38]. Higher call volumes persist, and anecdotal evidence suggests a shift from information-seeking calls about dementia to calls primarily from distressed carers and family members. This highlights the need to ensure responses are TI, recognizing that callers may be affected by trauma.

This exploratory study examined the type and nature of calls to the United Kingdom’s only 24‑hour dementia helpline and the extent to which call handlers’ responses reflected the TI principles of safety, trust, choice, collaboration, and empowerment. As the first study to investigate how TI approaches may be operationalized in this context, it provides preliminary insight into practical application within a population at heightened risk of trauma and identifies considerations for embedding these principles in future service design.

## Methods

### Study Design

This study used a retrospective mixed methods observational design, involving secondary analysis of routinely collected call handler logs from a national dementia helpline. A deductive framework approach was used to qualitatively examine the extent to which call handlers’ responses reflected predefined TI principles, alongside descriptive quantitative analysis of caller characteristics, call timing, reasons for contact, and theme frequency.

Reporting followed the Standards for Reporting Qualitative Research guideline [39], with items applied where relevant to secondary analysis of routinely collected service data. Items relating to participant recruitment, data saturation, and interview techniques were not applicable due to the retrospective evaluation design using anonymized call handler logs.

### Setting and Recruitment

No participants were actively recruited; instead, we used a purposeful sampling approach and focused on call logs collected between April 2020 and April 2021, a period when global COVID-19 restrictions were in place and demand for helpline support was markedly elevated. During this time, the Alzheimer Scotland helpline received 7357 calls, compared with an annual average of approximately 2000 calls. To select the study sample, Alzheimer Scotland applied a random number generation procedure to assign a random value to each unique call identifier and then retained the 200 logs with the lowest values. All identifiers were removed by Alzheimer Scotland prior to transferring the anonymized dataset to the research team. The research team had no contact with callers and no role in data generation or record selection.

Calls to the helpline are not audio recorded. Call logs completed by call handlers capture key caller concerns, callers’ emotions, and call handler response. Anonymized data capturing the call handler response were extracted for analysis in this retrospective study.

### Ethical Considerations

Ethics approval was obtained from the University of the West of Scotland Health and Life Sciences Academic Integrity and Ethics Committee (application reference 17641) for the secondary analysis of personal data. Organizational consent was granted by Alzheimer Scotland. The study involved analysis of deidentified, routinely collected call log records and did not involve direct contact with callers or helpline staff. As such, the project was classified as secondary use of personal data, and individual informed consent was not required. Prior to transfer, Alzheimer Scotland removed all direct and indirect identifiers and provided an anonymized dataset to the research team via secure transfer; data were then stored on secure servers at the University of the West of Scotland in compliance with institutional data governance and General Data Protection Regulation. The helpline does not record audio; only call handler summaries were available for analysis. No participants were recruited or compensated for this study. The manuscript and supplementary materials contain no identifiable images or media, and there is no risk of visual identification.

### Call Handler Training and Purpose of Logs

The helpline is staffed by volunteer call handlers who complete a 4‑day induction program focused on call handling skills and dementia information, followed by approximately 2 days of refresher training each year. Training includes awareness‑level TI content (eg, recognizing possible adversity or trauma and responding with empathy and validation) but did not include formal TI practice competency training during the study period. Call logs are used for documentation and operational follow‑up (eg, onward referral); we did not have access to a formal TI competency audit, and calls were not recorded, precluding verification against audio.

### Approach to Analysis

Retrospective document analysis [38] with framework analysis was used to organize and interpret the narrative summaries recorded by call handlers. An a priori framework matrix was used to chart the data according to the 5 TI principles of collaboration, empowerment, safety, choice, and trust. Quantitative methods analyzed demographic data from call logs on a range of caller characteristics (time of call, who is calling, diagnosis, and reason for call) and theme frequency. We report counts and percentages only. No inferential statistics were conducted due to small subgroup sizes, nonindependence of multiple themes per call log, and the subjective nature of theme coding.

### Qualitative Analysis

Framework analysis, guided by TI principles, was the conceptual framework for data analysis. The analysis was structured around the 5 TI principles defined by NHS Education for Scotland (NES) [40], which are explicitly aligned with the “Concept of Trauma and Guidance for a Trauma‑Informed Approach” of the US Substance Abuse and Mental Health Services Administrations [5]. These principles and their operational definitions were selected because they provide guidance that is directly appropriate to UK health and social care settings and well suited to the analysis of helpline interactions. The 5 principles applied were *safety* (ensuring physical and emotional safety with attempts to prevent retraumatization), *trust* (transparency, task clarity, and respecting interpersonal boundaries), *choice* (individual has meaningful choice, control, or voice in decision-making, with clear and appropriate message about rights), *collaboration* (recognizing the value of the caller experience, enabling decision-making, and providing knowledge to support the individual overcome challenges), and *empowerment* (sharing power, build capacity, and prioritize voice in decision-making with validation and affirmation of concerns).

Framework analysis is widely used as an effective analytic approach in multidisciplinary contexts and described as a pragmatic and rigorous data analysis technique [41]. A 5-stage process described by Ritchie and Spencer [42,43] (familiarization, identifying theme, indexing, charting and summarizing, and interpretation or mapping) was conducted by 3 members of the research team, with calibration checks performed by a fourth team member. In line with the recommendations on intercoder reliability in qualitative research [44], a sample of 10% (20/200) of call logs was reviewed by all members of the project team, with a very high level of agreement achieved (196/200, 98%). Call logs were divided between 3 reviewers for analysis, and the fourth team member reviewed a further sample from each group; a very high level of agreement was reported (198/200, 99%). Given the exploratory nature of this pilot, we report percentage agreement rather than κ and acknowledge its limitations. A thematic analysis was conducted using the framework analysis approach, enabling a structured and systematic examination of patterns across the dataset:

1. Familiarization involved reading each call log several times to understand the context of the call and how this was responded to by the call handler.
2. Identification of themes was defined in advance by the 5 NES TI principles of safety, trust, choice, empowerment, and collaboration.
3. Indexing involved applying the 5 NES themes to the data. Codes for themes were assigned at this stage.
4. Charting and summarizing involved the development of a matrix that mapped the data across the TI themes. Qualitative data were taken from Alzheimer Scotland call logs and inserted into a framework matrix in Microsoft Word [43], with columns representing each TI principle and rows containing illustrative quotations.
5. Interpretation of the TI framework allowed researchers to explore relationships within the data and generate insights that informed the development of the study narrative.

### Frequency of Themes

The systematic approach to coding the data supported the analysis of theme frequency. Theme frequency refers to how often each theme occurs across the dataset (reported as a percentage).

These data were analyzed using SPSS (version 25; IBM Corp) [45] to report demographic characteristics. Descriptive statistics report the frequency of each type of caller (eg, carer and person with dementia), time, reasons for the call, and occurrence of the TI themes. Independent samples 2-tailed *t* tests were initially considered to explore differences in theme frequency across caller subgroups; however, assumptions of normality and independence could not be verified given the sample size and nonindependent coding structure (multiple themes per call log). Consequently, inferential statistics were deemed inappropriate, and results are presented descriptively (counts and percentages) to avoid overstating findings.

## Results

### Qualitative Results

#### Overview

Qualitative results are presented thematically, with the most frequently occurring theme presented first. Collaboration was the most frequent theme, followed by empowerment, safety, choice, and trust. Illustrative data from the call logs are presented as blockquotes.

#### Collaboration

Among 179 calls made by carers, family members, or friends, collaboration was the most frequent theme (126/179, 70.4%) identified during daytime calls to the helpline and embodied the sharing of power and making decisions together, ensuring that the individual is at the center of planning and decisions. Call handlers demonstrated a collaborative drive to align people requiring support with local provision, for example, ensuring callers understood the role of the dementia adviser and how to contact them:

Gave her contact details and offered that I could email him.

Call log 73750861

Mentioned also Dementia Advisor and organisation such as Crossroads for befriender.

Call log 73963427

Sharing knowledge and strategies also involved electronic and hard copy information sources:

Please e-mail leaflets on Vascular Dementia; Let’s Talk about Dementia and Younger People with Dementia.

Call log 74360730

Call handlers approached the discussions around challenging aspects of dementia care with a collaborative problem-focused approach that aimed to share knowledge and resources and provide the caller with the skills and information to navigate the issue:

Caller said I had given her a lot to think about. Advised we are here 24/7. Call ended.

Call log 73959859

Suggested caller download “Worried about your Memory” and show mother or discuss it with her.

Call log 74360309

Talked about ways of encouraging mother to eat.

Call log 74360499

#### Empowerment

Linking the themes of “choice” and “collaboration” is the underpinning value of empowerment, which was noted in 60.3% (108/179) of calls. Call handlers demonstrated empowerment via information sharing but with a particular focus on delivering it in an encouraging and motivational manner that recognized the sensitivity around a dementia diagnosis:

Caller is clear that she needs to take some action and will call the AS resource in the first instance.

Call log 73957778

Caller would contact her GP and discuss these issues now she felt a little more confident about this.

Call log 74390087

Encouraged the caller to try and get help as she is finding it very hard at the moment to cope.

Call log 74385857

A TI approach, which recognizes that individuals may be impacted by trauma, was reflected within call handler responses that adopted an empowering approach. This was evidenced via recognition of the challenges expressed by callers noted within call logs, with call handlers offering reassurance and emotional support and explicitly highlighting an empathic approach to validating the experiences of those contacting the helpline. This implied that call handlers recognized the need to take the caller’s potential for distress into account during conversations while empowering them during the exchange:

Empathy. Reassured that caller is doing the best she can in very difficult circumstances.

Call log 74363829

Really distressed at making the call and not sure if she was doing the right thing—assured she was.

Call log 74385557

Validated client’s feelings of distress.

Call log 75632267

#### Safety

The theme of safety refers to the emotional, psychological, and physical safety demonstrated by call handlers and was identified in 58.7% (105/179) of calls. Call handlers addressed caller concerns regarding the health and safety of individuals with dementia, including worries about behaviors that might place either the person with dementia or the caller at risk, such as aggression or periods of isolation. They offered emotional support and reassurance in ways that conveyed empathy and understanding, acknowledging the unique difficulties faced by caregivers:

Mum going into a lovely care home in July. But when do they tell her? Talked about there being no right way.

Call log 73959312

During out-of-hours calls, “safety” emerged as the most common focus of call handlers’ responses. By validating callers’ emotions and recognizing the psychological stress involved, call handlers demonstrated an understanding of the distress caregivers experience:

To look after themselves because they themselves will be stressed by this process.

Call log 73959312

Call handlers also provided practical guidance and ongoing support, addressing unresolved issues and offering additional resources:

I feel caller would benefit from a call back as it felt like there were unresolved issues...caller not knowing where husband was etc.

Call log 74363829

As a last resort, if caller is concerned regarding friend’s welfare, discussed contacting the police.

Call log 73959859

For callers with dementia, call handlers offered emotional support tailored to their needs, alongside dementia-focused health information:

My main support was actually just to listen to her and she thanked me for being so patient.

Call log 72437207

#### Choice

Call logs reflected that call handlers engaged with callers using supportive, collaborative dialogue that reflected the issue of choice. They provided information about options for referrals and service signposting, ensuring callers had the information needed to make informed choices about their next steps (recorded in 67/179, 37.4% of calls):

I gave her option of doing it herself or me passing on the details. She said it will be one less thing for her to do if I do it.

Call log74362754

By informing callers of their rights and available services, call handlers supported decision-making across various domains of health and social care related to dementia and caregiving:

Discussed she is entitled to Adult Carer Support Plan. Husband also can be assessed. Discussed role of social services generally in this.

Call log 74362754

Support around legal matters was also provided:

Caller has Power of Attorney for sister so suggested that minor repairs out of sister’s funds are fine as long as they are in sister’s interest and careful receipt and record keeping maintained.

Call log 74364058

Stated that I was unsure of legal position as Mum’s home will be unoccupied as this is not her brother’s home and I would ask for legal position to be clarified and come back to caller.

Call log 75623115

There was considerable overlap between themes; for example, discussions related to both choice and trust, with call logs indicating awareness of the potential for distress in interactions with people who may be affected by dementia or concerned about symptoms:

Not wanting to bombard her with too much information and make her anxiety worse, I suggested she first call her practice to make a telephone appt. to speak to her GP...I told her about PDS in Scotland and gave her the Dementia Advisors contact details for her area.

Call log 72438646

I wonder whether the caller had actually received a diagnosis but has not remembered. However, I suggested that she get in touch with her GP...but as I wasn’t sure she had a formal diagnosis I didn’t want to overload or worry her more.

Call log 73132002

#### Trust

Trust was demonstrated implicitly through the rapport call handlers built to establish credibility while respecting professional boundaries and was identified in 31.3% (56/179) of calls. Call handlers emphasized the credibility of the organization and showed a commitment to addressing themes raised by callers in a compassionate manner:

I assured her that AS as an organisation does pick up on themes coming through and will take them forward when appropriate.

Call log 73749459

Call handlers also followed up with callers as needed, ensuring specialized support was provided, which demonstrated a commitment to meaningful engagement:

Returned call and informed caller that I would request a further return call to advise on legal position. Please contact caller and advise accordingly. PS Would also like to be informed on guidance given.

Call log 75623115

Empathy was a central aspect of building trust, as call handlers discussed sensitive topics with understanding and reassured callers of the helpline’s confidentiality:

Assured caller about confidentiality of Helpline along with telling him we could not give diagnosis.

Call log 76702833

Callers often expressed appreciation for the call handler’s balanced perspective and sensitivity, which reinforced their trust while ensuring that boundaries were maintained:

I did say that obviously I’m only hearing his perspective and his views, and he totally appreciated that too.

Call log 76703065

### Quantitative Results

The quantitative results provide data on caller characteristics, including the reason for call, caller identity, time of the call, and diagnosis. Differences in the frequency of themes in call handlers’ responses depending on the “time of call” and “caller identity” were also analyzed.

#### Reason for Call

There are 23 preidentified categories in the Alzheimer Scotland call log system indicating the reason for the call as assessed by the call handler. Call logs often recorded multiple reasons within a single call; the most common was “emotional support,” recorded 91 times across all call logs. This was followed by “carer stress” (66 instances) and “information on caring” (51 instances).

#### Caller Identity

Most of the 200 calls made to the helpline were from a “carer, family, or friend” (n=179, 89.5%). People with dementia calling the helpline about themselves (“self with dementia”) accounted for 6% (n=12) of calls, and people without a dementia diagnosis (“self-worried”) accounted for 3.5% (n=7) of calls. Callers who contacted the helpline about themselves are combined for the purposes of data analysis reported below. The remaining callers were “professionals” (n=2, 1%); these were not included in the analysis due to the small sample size; therefore, the dataset size is 198.

In 78% (n=155) of cases, there was a confirmed dementia diagnosis, while for 22% (n=45) of calls, a dementia diagnosis was not confirmed.

#### Caller Identity vs Theme Frequency

Exploration of differences in the call handler responses dependent upon the type of caller, that is, “carer, family, or friend” vs “self with dementia and/or self-worried” was carried out, with results reported in Table 1.

**Table 1:** Frequency of trauma-informed (TI) themes across caller types in 198 anonymized call logs (UK Alzheimer Scotland 24‑hour helpline; April 2020 to April 2021) using retrospective framework analysis^a^.

TI theme	Carer, family, or friend (n=179), n (%)	Self with dementia and self-worried (n=19), n (%)
Collaboration	126 (70.4)	14 (73.7)
Empowerment	108 (60.3)	14 (73.7)
Safety	105 (58.7)	13 (68.4)
Choice	67 (37.4)	6 (31.6)
Trust	56 (31.3)	6 (31.6)

^a^Multiple themes could be coded per call, so column percentages exceed 100%. Six cases had missing data.

Collaboration was the most frequent theme in the call logs of carers, family, or friends, followed by empowerment and safety. For people with a dementia diagnosis or worried about symptoms (self with dementia and self-worried), collaboration and empowerment were the most frequent themes, followed by safety. Choice and trust were also evident but were reported less frequently in this dataset.

#### Time of Call and Theme Frequency

The data on call times were recoded for analysis (Table 2). Most calls (159/198, 80%) were made during the day, between 6 AM and 5:59 PM, while the remaining 20% (39/198) were received at night, between 6 PM and 5:59 AM (Table 2).

**Table 2:** Distribution of call times for the 198 anonymized helpline call logs (UK Alzheimer Scotland 24‑hour helpline; April 2020 to April 2021), presented with the original 4 categories (morning, afternoon, evening, early hours) and a recoded day or night variable (day: 6 AM-17:59 PM; night: 6 PM-05:59 AM)^a^.

Time of call			Calls, n (%)
Original coding			
	Morning (6 AM-11:59 AM)	80 (40)	
	Afternoon (noon-6 PM)	79 (39.9)	
	Evening (after 6 PM-midnight)	31 (15.6)	
	Early hours (midnight-5:59 AM)	8 (4)	
Recoded			
	Daytime (6 AM-5:59 PM)	159 (80)	
	Nighttime (6 PM-5:59 AM)	39 (20)	

^a^Total missing values: n=2.

#### Call Time vs Theme Frequency

Table 3 presents the findings on theme frequency for daytime and nighttime calls. During the day (6 AM-5:59 PM), collaboration was the most frequently reported theme (112/159, 70.4%), and at night (6 PM-5:59 AM), safety was the most frequently reported theme (30/39, 76.9%). These differences in thematic profiles suggest a shift in the content of calls during the evening and early hours, with safety becoming a predominant focus in call handlers’ responses in the early hours.

**Table 3:** Frequency of trauma‑informed (TI) themes by recoded call time (day: 6 AM-05:59 PM; night: 6 PM-05:59 AM) for the 198 anonymized call logs from the UK Alzheimer Scotland 24‑hour dementia helpline (April 2020 to April 2021)^a^.

TI theme	Daytime (6 AM-5:59 PM; n=159), n (%)	Nighttime (6 PM-5:59 AM; n=39), n (%)
Collaboration	112 (70.4)	26 (66.7)
Empowerment	99 (62.3)	24 (61.5)
Safety	89 (56)	30 (76.9)
Choice	58 (36.5)	16 (41)
Trust	45 (28.3)	17 (43.6)

^a^Safety‑related responses were more common at night (30/39, 76.9%) than during the day (89/159, 56%); multiple themes could be coded per call.

## Discussion

### Overview

This pilot exploratory study of call handler summaries from the United Kingdom’s only 24‑hour dementia helpline suggests that responses frequently reflected TI principles, particularly collaboration and empowerment, with safety more evident during out‑of‑hours calls. Illustrative excerpts indicate empathetic listening, validation, shared problem‑solving, and signposting consistent with TI principles. Given the retrospective nature of the data and bias associated with this type of call logs, findings should be interpreted cautiously and considered hypothesis‑generating.

Because call logs rather than recorded calls were analyzed, the study reflects call handler interpretations rather than verbatim interactions. While our deductive framework ensured alignment with the study aim, it may have constrained identification of inductive themes. Therefore, we emphasize description over explanation and avoid claims about effectiveness.

### Principal Results

The prominence of collaboration and empowerment aligns with the helpline’s role as a gateway to support and information, while the higher frequency of safety‑oriented responses during evening and early hours is consistent with greater perceived vulnerability and limited access to routine services at those times. These patterns warrant prospective confirmation using richer data sources.

Reliable, accessible information for carers and people with dementia remains a major gap across the diagnostic pathway [46]. The helpline helped address this need by providing timely information and signposting, with “information on caring” and “difficulty accessing services” among the most common reasons for contact. In fragmented care systems that often fail to meet complex needs [46], carers frequently become what Brodaty and Donkin [17] term the “invisible second patient,” facing multiple inequalities and psychological distress without adequate skills or support.

Consistent with prior evidence on the high stress of dementia caregiving, “emotional support” was the most common reason for contacting the helpline, followed by “carer stress.” Responses reflected multiple TI principles, suggesting awareness of potential distress during exchanges [47]. Notably, 22% (45/200) of callers sought help without a confirmed diagnosis, indicating the helpline may serve as an early point of contact for individuals concerned about their own symptoms or those of a loved one [27].

### Strengths and Limitations

A strength of this study was the use of a predefined TI framework to guide analysis, which provided a systematic structure aligned with the aim. The stepwise approach to framework analysis and team‑based coding enhanced consistency and transparency, while interrater reliability checks increased confidence in coding validity.

However, several limitations must be acknowledged. First, the analysis relied on call handler summaries rather than recorded calls, introducing subjectivity and interpretive bias, as no audio verification was possible. The level of detail in call logs varied, which may reflect differences in call length or documentation practices. Volunteer call handlers had awareness-level TI training, but no formal competency audit was conducted, and retrospective document analysis carries inherent bias related to the original purpose of the logs. Findings also reflect a unique pandemic period and may not generalize beyond this context. Quantitative outputs were descriptive only, as inferential testing was not undertaken due to unmet assumptions. Finally, the impact of frequent exposure to distressed callers on call handlers was not assessed; future research should explore this using a TI lens.

### Implications and Future Research

This pilot exploratory study highlights the potential value of embedding TI principles within helpline services for people affected by dementia. Services may consider structured TI training, routine reflective supervision, and feasible quality assurance measures such as periodic recording (with consent) and documentation audits to support fidelity and staff well-being. Future research should triangulate call logs with recorded calls and caller‑reported outcomes, use validated coding protocols, and examine the impact of empathic engagement on call handlers. Prospective studies using richer data sources and robust designs are needed to confirm these preliminary findings and inform best practice in TI helpline provision.

### Conclusions

This pilot exploratory study examined call handler responses from the United Kingdom’s only 24‑hour dementia helpline to assess the extent to which TI principles were reflected in practice. Findings suggest that, despite only brief awareness‑level training, call handlers demonstrated approaches consistent with TI principles, including collaboration, empowerment, and safety.

To advance international aspirations for TI care, helpline services for dementia and other life‑altering conditions should consider embedding structured TI training, reflective supervision, and safeguards to minimize the risk of trauma for both callers and volunteers. This is particularly important as volunteers may experience cumulative exposure to distressing information.

Although limited by reliance on call logs and the unique pandemic context, this study provides preliminary evidence that TI principles can be operationalized within helpline interactions. Future research should confirm these findings using recorded calls, caller‑reported outcomes, and robust evaluation designs to inform best practice for TI helpline provision.

## Data Availability

The data analyzed in this study comprise an anonymized subset of routinely collected operational helpline call logs owned by Alzheimer Scotland. The data were accessed under organizational permission for the purposes of this evaluation only. Due to restrictions imposed by the data owner, together with the residual risk of reidentification inherent in narrative call summaries, the underlying individual-level data are not publicly available and cannot be shared.

## Abbreviations

NES: NHS Education for Scotland
TI: trauma-informed
